# Polygenic scores for longitudinal prediction of incident type 2 diabetes in an ancestrally and medically diverse primary care physician network: a patient cohort study

**DOI:** 10.1186/s13073-024-01337-0

**Published:** 2024-04-26

**Authors:** Ravi Mandla, Philip Schroeder, Bianca Porneala, Jose C. Florez, James B. Meigs, Josep M. Mercader, Aaron Leong

**Affiliations:** 1 Programs in Metabolism and Medical and Population Genetics, Broad Institute of MIT and Harvard, Cambridge, MA USA; 2 Diabetes Unit, Endocrine Division, Department of Medicine, Massachusetts General Hospital, Boston, MA USA; 3 Center for Genomic Medicine, Massachusetts General Hospital, Boston, MA USA; 4 Division of General Internal Medicine, Department of Medicine, Massachusetts General Hospital, 100 Cambridge St. Fl. 16, Boston, MA 02114 USA; 5 Department of Medicine, Harvard Medical School, Boston, MA USA

**Keywords:** Polygenic scores, Type 2 diabetes, Primary care, Electronic health records

## Abstract

**Background:**

The clinical utility of genetic information for type 2 diabetes (T2D) prediction with polygenic scores (PGS) in ancestrally diverse, real-world US healthcare systems is unclear, especially for those at low clinical phenotypic risk for T2D.

**Methods:**

We tested the association of PGS with T2D incidence in patients followed within a primary care practice network over 16 years in four hypothetical scenarios that varied by clinical data availability (*N* = 14,712): (1) age and sex; (2) age, sex, body mass index (BMI), systolic blood pressure, and family history of T2D; (3) all variables in (2) and random glucose; and (4) all variables in (3), HDL, total cholesterol, and triglycerides, combined in a clinical risk score (CRS). To determine whether genetic effects differed by baseline clinical risk, we tested for interaction with the CRS.

**Results:**

PGS was associated with incident T2D in all models. Adjusting for age and sex only, the Hazard Ratio (HR) per PGS standard deviation (SD) was 1.76 (95% CI 1.68, 1.84) and the HR of top 5% of PGS vs interquartile range (IQR) was 2.80 (2.39, 3.28). Adjusting for the CRS, the HR per SD was 1.48 (1.40, 1.57) and HR of the top 5% of PGS vs IQR was 2.09 (1.72, 2.55). Genetic effects differed by baseline clinical risk ((PGS-CRS interaction *p* = 0.05; CRS below the median: HR 1.60 (1.43, 1.79); CRS above the median: HR 1.45 (1.35, 1.55)).

**Conclusions:**

Genetic information can help identify high-risk patients even among those perceived to be low risk in a clinical evaluation.

**Supplementary Information:**

The online version contains supplementary material available at 10.1186/s13073-024-01337-0.

## Background

More than a fifth of outpatient visits are for patients with diabetes, largely type two diabetes (T2D), placing a heavy burden on US healthcare systems [1, 2]. Identifying individuals with elevated risk for T2D is crucial to channel resources towards those most likely to benefit from interventions that prevent or delay T2D and its complications [3]. To assess clinical risk factors for T2D (e.g., family history of T2D, obesity, hypertension, hyperlipidemia), clinicians perform a clinical evaluation and order fasting laboratory (lab) tests (e.g., fasting glucose and lipid panel) [4, 5]. However, patients who do not engage frequently with the healthcare system or are perceived to be low risk at a routine health maintenance exam may not have regular lab testing or follow-up visits, limiting accurate risk estimation.

Genetic information, if available, could improve T2D prediction among patients lacking measured clinical risk factors [6]. Genome-wide association studies (GWAS) have identified hundreds of unique loci associated with T2D [7], the results of which can be used to calculate polygenic scores (PGS) that model genetic risk independently of established clinical risk factors including family history [8, 9]. Previous work has evaluated how PGS can be used within healthcare systems [6, 10], but analyses have been largely cross-sectional in biobanks of mostly European ancestry, limiting the generalizability of results to a more ancestrally and medically diverse US healthcare system. While PGS have been shown to only modestly improve prediction over traditional clinical risk factors [11, 12], the long-term prediction of T2D by PGS in clinical scenarios that vary in clinical information availability in medical records has not been well studied.

In a large academic primary care physician (PCP) network affiliated with Mass General Hospital (MGH) [13, 14], some patients had volunteered to be genotyped in the Mass General Brigham (MGB) Biobank. We hypothesized that PGS constructed from their genetic data would be associated with incident T2D over a follow-up period of up to 16 years even after adjusting for clinical risk factors available in their electronic health records (EHR). We further hypothesize that PGS would have the most added predictive value in patients with sparse baseline clinical data at the time of their initial clinical encounter (i.e., risk factors such as random glucose have not been measured or captured in the EHR).

We constructed four nested cohorts based on hypothetical scenarios by which a patients with genetic data might interact with the healthcare system: (1) a person registered themselves as a patient and provided demographic data; (2) a patient had a visit with a healthcare provider during which their weight, height, and blood pressure were measured and a medical history was taken; (3) a patient had a random glucose measured from a basic lab panel in addition to having a visit with a healthcare provider; (4) a lipid panel, which usually requires the patient to fast overnight, was also performed enabling calculation of the Framingham T2D clinical risk score (CRS) [15]. We generated Cox models adjusted for baseline clinical variables in each of these scenarios and evaluated improvements to T2D incidence prediction when including PGS. We then performed stratified analyses by age, body mass index (BMI), random glucose, and CRS to evaluate the additional prediction information of PGS for patients that would be considered “lower risk” or “higher risk” at baseline.

## Methods

### Study aim, design and setting

We sought to evaluate the longitudinal predictive performance of T2D PGS for incident T2D by creating simulated scenarios that varied in depth of clinical data availability at the time of each patient’s initial clinical encounter in the MGH PCP network. We extracted two decades of clinical data for 284,602 patients who had received primary care through the network from an EHR repository including outpatient, emergency department, and inpatient visits. Patients were eligible to be in the study cohort if they had at least two clinical encounters between January 1, 2000, and December 31, 2020, and available genetic data through the MGB Biobank (*N* = 15,355). The MGB Biobank is a large biorepository with genetic data for over 50K participants aged 18 years or older who had been recruited from affiliated hospitals, clinics, or cohorts or provided electronic informed consent [16]. After excluding patients with a T2D diagnosis before or within 6 months of their first encounter, 14,712 patients remained for downstream analysis. The MGB institutional review board approved the study.

### Clinical characteristics of participants

Sociodemographic variables included baseline age, self-reported race/ethnicity, educational attainment, and gender. Baseline clinical diagnoses for coronary heart failure, peripheral vascular disease, proteinuria, and hypertension (HTN) were defined with ICD-10 code-based algorithms between January 1, 2000, and up to 6 months after the initial encounter. T2D [17], coronary artery disease (CAD), and chronic kidney disease (CKD) duration at baseline was calculated from the diagnosis date to the date of the initial encounter. Mean lab values were extracted between 18 months before the initial encounter and 6 months after the initial encounter. For BMI, we used any available BMI measured during the follow-up period as repeated measurements exhibited low variability (median standard deviation of BMI: 0.95) and very few BMI measurements were available in that two-year window.

### Calculation of T2D CRS

We implemented the Framingham T2D CRS, comprising age, sex, parental history of T2D, BMI, systolic blood pressure (SBP), high-density lipoprotein (HDL), total cholesterol, triglyceride, fasting glucose, and waist circumference [15]. We used family history as a proxy for parental history. As waist circumference and fasting status for glucose were not available, both variables were set as constants in the CRS, and random glucose was included as an independent variable. The CRS was then log-transformed to be normally distributed, and standardized. The full CRS implemented can be found in the Supplementary Methods (Additional file 1).

### Outcome

The start of observation time was January 1, 2000, or 6 months after the first clinical encounter if it occurred after January 1, 2000. Incidence of T2D was defined with ICD-10 code-based algorithms (Additional file 2: Table S1) during the observation time and right-censored at initial disease occurrence or the last clinical encounter before December 15, 2021.

### Genotyping data preparation

Genotypes were measured using either the Illumina Multi-Ethnic Genotyping Array (MEGA) or the Illumina Global Screening Array (GSA). We accessed MEGA genotyping data for 36K participants and GSA genotyping data for 18K participants from the MGB Biobank then processed by batch. Briefly, the quality control steps included filtering out variants based on minor allele frequency (MAF) levels (< 5%), missingness (*>* 0.05), genotyping batch bias (*P* < 5 × 10^−5^), and Hardy-Weinberg equilibrium (*P* < 1 × 10^−10^) and palindromic single nucleotide variants (AT or CG). Individuals were also removed if their self-reported sex did not match their genetic sex or if they had a high ratio of heterozygote variants. These clean datasets were phased using Shapeit4, imputed using the TOPMed *r*2 reference panel, then union merged. Quality control, phasing, and imputation steps were not stratified by self-reported race subgroups except for variant filtering based on Hardy-Weinberg equilibrium and participant filtering based on heterozygosity.

To calculate Principal Components (PCs) and genetic ancestry probabilities for individuals in MGB Biobank, we first created an intersection of common (MAF > 5%, genotyping rate > 0.95%), independent (*R*2 < 0.1) variants from both the HGDP/1000G dataset and MGB Biobank. Next, PCs were calculated among the HGDP/1000G dataset and MGB Biobank was projected into this PC space. Genetic ancestry probabilities were calculated using a random forest classification model, trained on PCs and continental ancestry data from HGDP/1000G and applied to MGB Biobank. In our analysis, we assigned an individual to a continental ancestry if that genetic ancestry probability was > 0.5.

### Calculation of T2D PGS

We selected large GWAS meta-analyses for our traits of interest of which full summary statistics were available for the calculation of PGS. For T2D we meta-analyzed the published MVP/DIAMANTE meta-analyses results with T2D GWAS from the FINNGEN Biobank r6 [18, 19]. Genome-wide PGS were calculated using PRScs with the provided EUR 1000G HapMap3 LD reference files [20]. Posterior weights from PRScs were used to calculate the PGS in the MGB biobank with the PLINK --score function. To account for PGS variability in our multi-ancestry cohort, we implemented a modified PGS adjustment strategy based on previously published methods [10, 21]. Briefly, we fitted a linear model of each disease-specific PGS against genetic ancestry probabilities. Adjusted PGS were calculated as the residual between the predicted and actual PGS in the entire dataset.

### Primary statistical analyses

We tested the association of PGS with T2D incidence in Cox models adjusted for the available clinical variables in each scenario, including all participants in the primary analysis regardless of ancestry, and corrected for 10 PCs in all models to account for population stratification. As a sensitivity analysis, we converted the PGS into a categorical variable to compare individuals of high genetic risk based on a percentile cutoff to those of average genetic risk based on the interquartile range (IQR) within each scenario. We explored three different high-risk cutoffs: 5%, a less stringent 10%, and a more stringent 2% which has been suggested for T2D [22]. For purposes of model comparison, we chose 5% as the primary analysis due to being a middle ground between high genetic risk and sample size drop offs when selecting a cutoff value. Kaplan-Meier curves and Cox models were generated using the lifelines package in Python [23]. The estimated probability of incident T2D was calculated as the predicted probability of developing T2D during the follow-up time. Logistic regression models were fit with T2D developed during the follow-up as the response variable and all available clinical risk factors of a specific scenario, the T2D PGS, and the first 10 PCs as predictor variables. These were the same predictor variables used in the combined clinical and PGS Cox models. Models were trained then applied on all participants within a scenario. Tertiles of PGS were defined among participants within each scenario.

We calculated the change in model performance using the c-index [24] upon adding PGS to several clinical base models: scenario 1 age and sex; scenario 2 age, sex, family history of T2D, BMI, and systolic blood pressure (SBP); scenario 3 age, sex, family history of T2D, BMI, SBP, and random glucose; scenario 4 age, sex, family history of T2D, BMI, SBP, HDL, total cholesterol, and triglycerides combined into a clinical risk score (CRS) and random glucose as an independent variable. We evaluated the performance of the full CRS in our models using the pre-computed, published weights for each individual variable from the Framingham T2D CRS [15]. Full formulas for the cox models used in each scenario can be found in the Supplementary Methods (Additional file 1).

In a sensitivity analysis, we created separate models built on individual variables without using pre-computed CRS weights in our complete cases. In another sensitivity analysis, we imputed missing lab values (max of 8% total missing) for individuals missing only one lab (HDL, cholesterol, triglycerides, or glucose) using multivariate feature imputation, imputing missing values using predictions modelled on all available clinical risk factors.

For scenario 2, scenario 3, and the imputation sensitivity analysis in scenario 4, BMI values were log-transformed to be normally distributed. Outlying BMI values, defined as those with a log BMI value more than 4 standard deviations away from the mean log BMI value, were also removed. For scenario 3 and all scenario 4 analyses, glucose was double log-transformed to be normally distributed. For scenario 3 and the imputation sensitivity analysis in scenario 4, triglycerides were log-transformed.

### Stratified statistical analyses

For each scenario, we tested for significant statistical interaction between the PGS and the clinical variable available in each scenario that was most associated with T2D, adjusting for all available variables per scenario. We then stratified our analyses by the clinical variable available in each scenario that was most associated with T2D. For scenario 1, we tested for an interaction between PGS and a binary age cutoff, and stratified by the recommended age cutoff from the ADA and CDC to commence screening for T2D at routine health examinations [25, 26], 40 years. For scenario 2, we tested for an interaction between PGS and log-transformed BMI and stratified by the median BMI of our dataset (27.5 kg/m^2^). For scenario 3, we tested for an interaction between PGS and double log-transformed random glucose and stratified by a random glucose cutoff of 100 mg/dL (threshold for impaired fasting glucose, presuming blood tests were drawn fasting). For scenario 4, we tested for an interaction between PGS and log-transformed CRS and stratified by the median CRS value.

### PGS prediction of CKD and CAD as T2D-related complications

As CKD and CAD are two leading causes of death in people with T2D [27, 28], we tested whether PGS can improve prediction of incident CAD and CKD over CRS. For CKD we used the SCORED CRS, which required age, sex, and diagnoses of anemia, HTN, T2D, CHD, congestive heart failure, peripheral vascular disease, and proteinuria diagnoses [29]. The equation used to calculate the CKD CRS can be found in the Supplementary Methods (Additional file 1). For CAD we used the Framingham CAD CRS, which required age, sex, smoking, total cholesterol, HDL measurements, SBP, and HTN treatment [30]. We used HTN diagnosis as a substitute for HTN treatment. The equation used to calculate the CAD CRS can be found in the Supplementary Methods (Additional file 1).

We considered two scenarios for these analyses: (1) a clinical visit without labs and (2) a clinical visit with labs. For CAD, the clinical variables in each scenario were (1) age, sex, smoking status, and SBP and (2) age, sex, smoking status, SBP, HDL, and total cholesterol combined into a CRS. The Cox model formulas used for CAD incidence can be found in the Supplementary Methods (Additional file 1). For CKD, the clinical variables per model (1) age, sex, diagnosis history, SBP, diastolic blood pressure (DBP), weight, and HTN, and (2) the risk factors from (1) and anemia status determined by hemoglobin count combined into a CRS. The Cox model formulas used for CKD incidence can be found in the Supplementary Methods (Additional file 1).

Using the PRScs method, we constructed CKD and CAD PGS with the summary statistics from the CKD Gen Consortium [31], and Nelson et al. of UK Biobank SOFT CAD GWAS with CARDIoGRAMplusC4D 1000 Genomes-based GWAS and the Myocardial Infarction Genetics and CARDIoGRAM [32]. We then generated incidence models using the pertinent clinical variables from their respective CRS available by T2D status.

## Results

We compared the demographic characteristics of genotyped patients in the PCP network (*N* = 15,355), non-genotyped patients (*N* = 269,247) in the network, and genotyped patients in the MGB biobank but not in the network (*N* = 38,107; Additional file 2: Table S2). Patients with genetic data within the PCP network had a higher proportion of T2D, higher proportion of self-reported non-Hispanic white individuals, and higher educational attainment compared to patients without genetic data and patients with genetic data but not in the network. The characteristics of genotyped patients in the PCP network were similar to those of the genotyped patients in the PCP network after excluded patients with T2D before the start date (*N* = 14,712).

Patients included in scenarios 3 and 4 with a greater number of clinical risk factors available for analysis at baseline were on average older, had a higher proportion of comorbidities, and a smaller proportion of current smokers compared with patients included in scenario 1 and scenario 2. Other baseline characteristics were similar across scenarios (Table 1).

**Table 1:** Patient characteristics in the MGH primary care physician network with genetic data by scenario

	**Scenario 1**	**Scenario 2**	**Scenario 3**	**Scenario 4**
*n*	14,712	13,670	9867	7331
Age, mean (SD)	48.9 (15.7)	48.9 (15.4)	51.3 (14.6)	53.9 (13.2)
Female, *n* (%)	7965 (54.1%)	7430 (54.4%)	5124 (51.9%)	3480 (47.5%)
Current smokers, *n* (%)	902 (6.1%)	766 (5.2%)	554 (3.8%)	352 (2.4%)
Self-Reported Race				
White, *n* (%)	12,697 (86.3%)	11,814 (86.4%)	8592 (87.1%)	6511 (88.8%)
Black/African American, *n* (%)	525 (3.6%)	492 (3.6%)	373 (3.8%)	267 (3.6%)
Asian, *n* (%)	330 (2.2%)	293 (2.1%)	187 (1.9%)	119 (1.6%)
Other/unknown, *n* (%)	1160 (7.9%)	1071 (7.8%)	715 (7.2%)	434 (5.9%)
Self-Reported Ethnicity				
Hispanic, *n* (%)	227 (1.5%)	185 (1.4%)	123 (1.2%)	80 (1.1%)
Non-Hispanic, *n* (%)	12,407 (84.3%)	11,502 (84.1%)	8294 (84.1%)	6139 (83.7%)
Other/unknown, *n* (%)	2078 (14.4%)	1983 (14.5%)	1450 (14.7%)	1112 (15.2%)
Genetic ancestry				
EUR, *n* (%)	12,508 (85.0%)	11,639 (85.1%)	8450 (85.6%)	6406 (87.4%)
AFR, *n* (%)	520 (3.5%)	489 (3.6%)	377 (3.8%)	269 (3.7%)
AMR, *n* (%)	1003 (6.8%)	924 (6.8%)	627 (6.4%)	357 (4.9%)
ASIAN, *n* (%)	357 (2.4%)	316 (2.3%)	202 (2.0%)	134 (1.8%)
Other, *n* (%)	324 (2,2%)	302 (2.2%)	211 (2.1%)	165 (2.3%)
Highest educational attainment				
High school, *n* (%)	3364 (22.9%)	3113 (22.8%)	2357 (23.9%)	1683 (23.0%)
Undergraduate, *n* (%)	6378 (43.4%)	5956 (43.6%)	4134 (41.9%)	3045 (41.5%)
Graduate, *n* (%)	2195 (14.9%)	2088 (15.3%)	1458 (14.8%)	1106 (15.1%)
Other/unknown, *n* (%)	2775 (18.9%)	2513 (18.4%)	1918 (19.4%)	1497 (20.4%)
Diagnoses at baseline				
Coronary artery disease, *n* (%)	713 (4.8%)	627 (4.6%)	573 (5.8%)	531 (7.2%)
Chronic kidney disease, *n* (%)	376 (2.6%)	321 (2.3%)	301 (3.1%)	264 (3.6%)
Hypertension, *n* (%)	3247 (22.1%)	3046 (22.3%)	2793 (28.3%)	2410 (32.9%)
Chronic heart failure, *n* (%)	207 (1.4%)	177 (1.3%)	162 (1.6%)	126 (1.7%)
Peripheral vascular disease, *n* (%)	136 (0.9%)	115 (0.8%)	103 (1.0%)	78 (1.1%)
Family history of T2D, *n* (%)	581 (3.9%)	555 (4.1%)	413 (4.2%)	309 (4.2%)
Family history of CAD, *n* (%)	4319 (29.4%)	4109 (30.1%)	3018 (30.6%)	2330 (31.8%)
Incident diabetes during follow-up, *n* (%)	1908 (13.0%)	1800 (13.2%)	1487 (15.1%)	1265 (17.3%)
Max follow-up time (years)	15.9	15.9	15.9	15.9
BMI, mean (SD)		28.3 (5.7)	28.5 (5.7)	28.7 (5.6)
Systolic blood pressure, mean (SD)		125.7 (11.8)	126.6 (11.7)	127.6 (11.3)
Diastolic blood pressure, mean (SD)		75.0 (6.7)	74.9 (6.6)	74.9 (6.5)
Random glucose, mean (SD)			97.2 (22.9)	96.9 (22.0)
Total cholesterol, mean (SD)				189.9 (35.9)
HDL, mean (SD)				56.5 (17.1)
Triglyceride, mean (SD)				128.2 (86.6)

Follow-up time was calculated as the length of time between the first diagnosis of diabetes and either January 1, 2000, or a patient’s first clinical encounter into the PCP. Data was left-censored to remove participants with a diagnosis of T2D before their start date. Only complete cases for all clinical risk factors included in each scenario were used in the primary analyses. Clinical risk factors at the time of first encounter in each scenario are as follows: scenario 1 age, sex; scenario 2 age, sex, BMI, family history of T2D, systolic blood pressure; scenario 3 age, sex, BMI, family history of T2D, systolic blood pressure, random glucose; scenario 4 age, sex, BMI, family history of T2D, systolic blood pressure, triglycerides, total cholesterol, and HDL combined into a clinical risk score and random glucose

Kaplan-Meier curves demonstrated full separation of PGS tertiles among all patients and in the subset of patients of European ancestry, who are most genetically similar to the cohorts used to derive the T2D PGS (Additional file 3: Fig. S1A, S1B). However, among the patients not of European ancestry, we observed poorer separation of PGS tertiles possibly due to both smaller sample sizes and allele frequency differences in this heterogeneous population (Additional file 3: Fig. S1C). Adjusting T2D PGS by genetic ancestry probabilities (see the “Methods” section) improved separation of PGS tertiles for this group (Additional file 3: Fig. S1D, S1E, and S1F), and corrected bias caused by PGS distribution differences across genetic ancestries; thus, residualized, ancestry-adjusted PGS were used in all subsequent analyses and all patients were analyzed as a single cohort.

**Fig. 1:**
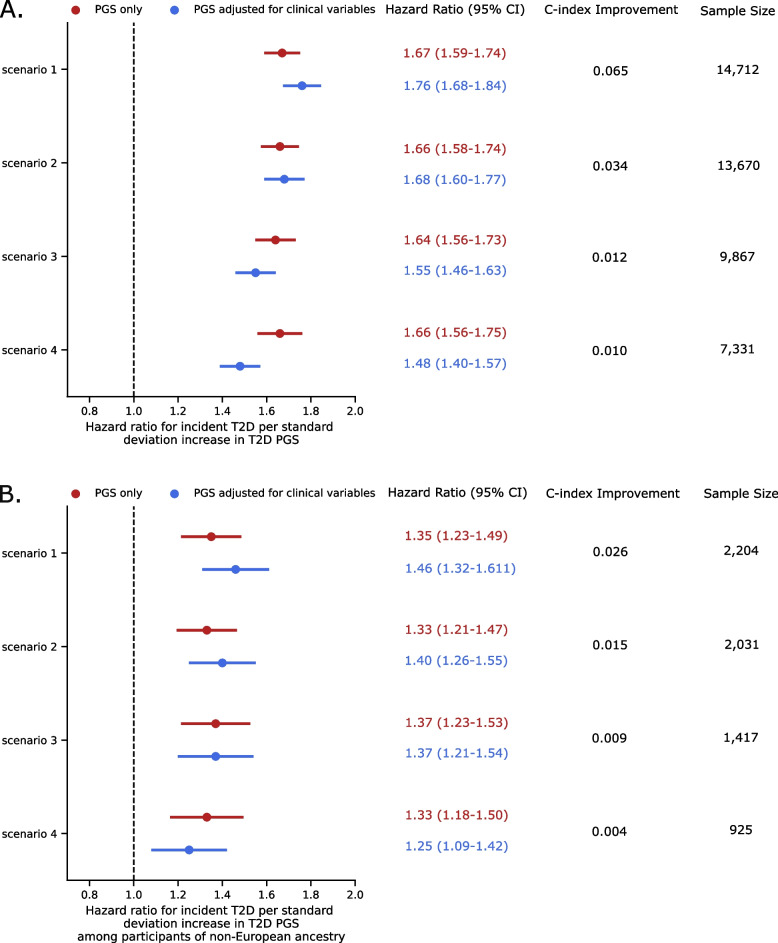
Association of T2D PGS with incident T2D with and without adjustment for clinical variables. In all four scenarios of clinical data availability, the T2D PGS provide additional predictive information on top of clinical risk factors based on T2D PGS HR adjusted for clinical risk factors (T2D PGS adjusted HR of 1.75 in scenario 1, 1.68 in scenario 2, 1.54 in scenario 3, and 1.47 in scenario 4) and c-index improvements of including T2D PGS in clinical risk models. These benefits are largest in scenarios of minimal data availability and is true both among (**A**) the total cohort and (**B**) participants of non-European ancestry only. Clinical risk factors in each scenario are as follows: scenario 1 age, sex; scenario 2 age, sex, BMI, family history of T2D, systolic blood pressure; scenario 3 age, sex, BMI, family history of T2D, systolic blood pressure, random glucose; scenario 4 age, sex, BMI, family history of T2D, systolic blood pressure, triglycerides, total cholesterol, and HDL combined into a clinical risk score and random glucose

T2D PGS was associated with incident T2D and had similar HR in all scenarios adjusting only for PCs (scenario 1 HR per SD of PGS: 1.67 (95% CI 1.59–1.74, *p* = 1.5 × 10^−104^) (Fig. 1A, Additional file 2: Table S3). The association was preserved even after adjusting for clinical variables available in each of the scenarios. Predictive performance improved in every scenario when adding PGS to the clinical risk variables model (LRT, *P* < 0.001) (Fig. 1A, Additional file 2: Table S3, Table 2). The improvement was most appreciable in scenario 1 with only age, sex, and PCs in the base model. The adjusted HR was 1.76 per SD of the PGS (95% CI 1.68–1.84; *P* = 1.1 × 10^−124^; c-index improvement: 0.065; Additional file 2: Table S3) and the adjusted HR of the top 5% of the PGS compared to the IQR was 2.80 (95% CI 2.39-3.28; *P* = 1.3 × 10^−37^; Table 2). In scenario 4, with more clinical variables in the base model, the adjusted HR was 1.48 per SD of the PGS (95% CI 1.40–1.57; *P* = 2.0 × 10^−39^; c-index improvement over base model: 0.01; Additional file 2: Table S3) and the adjusted HR of the top 5% of the PGS compared to the IQR was 2.09 (95% CI 1.72–2.55; *P* = 1.7 × 10^−13^; Table 2). The performance of the PGS was lower among the patients of non-European ancestries (Additional file 2: Table S3) likely because the PGS were derived from meta-analyses summary statistics with an overrepresentation of European descent. We conducted two sensitivity analyses for the clinical base model in scenario 4. First, we performed multiple imputation for missing lab values (< 8% missing). Second, we used the individual clinical variables without combining them in a CRS. The improvement of the c-index when adding PGS to the base clinical model was similar when using multiple imputation and when using individual clinical variables instead of the CRS (Additional file 2: Table S3).

**Table 2:** Association of polygenic scores as a categorical variable (top 5% vs IQR) with incident T2D

	**Scenario 1**	**Scenario 2**	**Scenario 3**	**Scenario 4**
***n* of T2D cases (*n* in top 5%, *n* in IQR)**	1073 (199, 874)	1002 (184,818)	827 (152, 675)	712 (132, 580)
**Total *n* (*n* in top 5%, *n* in IQR)**	8092 (736, 7356)	7518 (684, 6834)	5427 (494, 4933)	4032 (367, 3665)
**Clinical variables only model**				
C-index	0.675	0.752	0.816	0.801
CRS HR (CI) (*p*-val)				1.71 (1.56–1.88) (1.6e−29)
**PGS only model**				
C-index	0.608	0.607	0.603	0.608
PGS HR (CI) (*p*-val)	2.43 (2.08–2.85) (1.0e−28)	2.40 (2.04–2.83) (7.1e−26)	2.43 (2.03–2.91) (3.5e−22)	2.58 (2.13–3.14) (7.3e−22)
**Combined clinical and PGS model**				
C-index	0.702	0.769	0.822	0.803
CRS HR (CI) (*p*-val)				1.71 (1.56–1.88) (6.6e−29)
PGS HR (CI) (*p*-val)	2.80 (2.39–3.28) (1.3e−37)	2.65 (2.25–3.12) (3.3e−31)	2.40 (2.0–2.88) (4.9e−21)	2.09 (1.72–2.55) (1.7e−13)
**C-index improvement**	0.027	0.017	0.006	0.002
**LRT *p*-value**	8.77E−31	6.12E−26	5.44E−18	7.09E−12

Longitudinal models were constructed with either clinical variables included in each scenario, polygenic scores (PGS) only, or both the clinical variables and PGS in a combined model. PGS were converted into a categorical value differentiating participants in the top 5% of the PGS compared to those in the interquartile range (IQR) of the PGS. Clinical risk factors in each scenario are as follows: scenario 1 age, sex; scenario 2 age, sex, BMI, family history of T2D, SBP; scenario 3 age, sex, BMI, family history of T2D, SBP, random glucose; scenario 4 age, sex, BMI, family history of T2D, SBP, triglycerides, total cholesterol, and HDL combined into a clinical risk score (CRS) and random glucose. We reported the concordance index (C-index), hazard ratio (HR) for the PGS for being in the top 5% compared to the IQR of the PGS or CRS per standard deviation depending on if they are included in the model, and the log-likelihood ratio test (LRT) *p*-value from comparing the difference in performance between the combined clinical and PGS model with the clinical variables only model

We also explored how different PGS thresholds of risk associate with incident T2D in each scenario in addition to using a top 5% cutoff, including a top 10% cutoff and top 2% cutoff. When adding the PGS on top of clinical risk factors available in scenario 1 (age, sex, and PCs), the adjusted HR of the top 10% compared to the IQR was 2.52 (95% CI: 2.23–2.85, *P*: 6.6 × 10^−49^, c-index improvement over base model: 0.034; Additional file 2: Table S4) and the adjusted HR of the top 2% compared to the IQR was 3.52 (95% CI: 2.84–4.37; *P*: 4.9 × 10^−30^; c-index improvement over base model: 0.021; Additional file 2: Table S4). In scenario 4 when adjusting for the T2D CRS, random glucose, and PCs, the adjusted HR of the top 10% of the PGS compared to the IQR was 1.98 (95% CI 1.70–2.31; *P* = 1.9 × 10^−18^; c-index improvement over base model: 0.003; Additional file 2: Table S4) and the adjusted HR of the top 2% of the PGS compared to the IQR was 2.55 (95% CI 1.94–3.36; *P* = 1.8 × 10^−11^; c-index improvement over base model: 0.002; Additional file 2: Table S4).

We found statistical interaction (*P*_interaction_ < 0.05), between T2D PGS and the clinical risk factor most associated with incident T2D in scenarios 1, 2, and 3 (i.e., age, BMI, and random glucose), and a borderline interaction between T2D CRS and PGS in scenario 4 (*P*_interaction_ = 0.053), motivating stratified analyses by clinical risk factors (Additional file 2: Table S5). The PGS estimates were larger among individuals age < 40 years (HR 1.88; 95% CI 1.66–2.13; *n* = 4407; c-index = 0.73) vs. age ≥ 40 years (HR 1.74; 95% CI 1.65–1.82; *n* = 10,305; c-index = 0.70) (Additional file 2: Table S5), among individuals with BMI <27.5 kg/m^2^ (HR 1.78, 95% CI 1.62–1.97; *n* = 6930; c-index = 0.78) vs. BMI ≥ 27.5 kg/m^2^ (HR 1.65, 95% CI 1.56–1.74; *n* = 6740; c-index = 0.73) (Additional file 2: Table S5), among individuals with glucose < 100 mg/dL (HR 1.59; 95% CI 1.46–1.74; *n* = 6903; c-index = 0.79) vs. glucose > 100 mg/dL (HR 1.48; 95% CI 1.38–1.58; *n* = 2964; c-index = 0.77) (Additional file 2: Table S5), and with T2D CRS < median (HR 1.60; 95% CI 1.43–1.79; *n* = 3665; c-index = 0.82) vs. > median (HR 1.45; 95% CI 1.35–1.55; *n* = 3666; c-index = 0.78) (Additional file 2: Table S5).

**Fig. 2:**
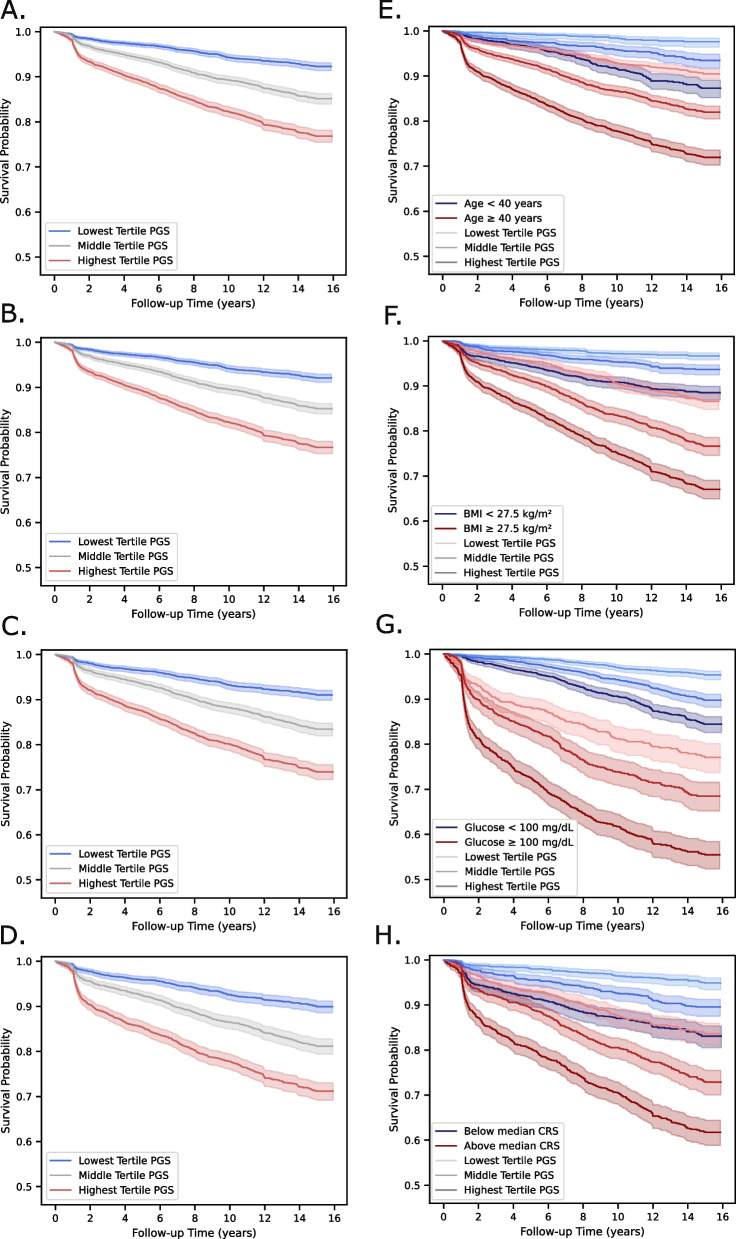
Kaplan-Meier curves with and without stratification by baseline clinical risk factors. T2D PGS tertiles show strong separation of T2D onset in (**A**) scenario 1, (**B**) scenario 2, (**C**) scenario 3, and (**D**) scenario 4. In each scenario, the multivariate log-rank test was *P* < 0.0001. T2D PGS tertiles further stratify risk over available clinical risk factors at each scenario, including (**E**) an age cutoff of 40 years in scenario 1, (**F**) a BMI cutoff of 27.5 kg/m^2^ in scenario 2, (**G**) a random glucose of 100 mg/dL in scenario 3, and (**H**) the median T2D CRS in scenario 4. In each stratification analysis per scenario, the multivariate log-rank test was *P* < 0.0001

**Fig. 3:**
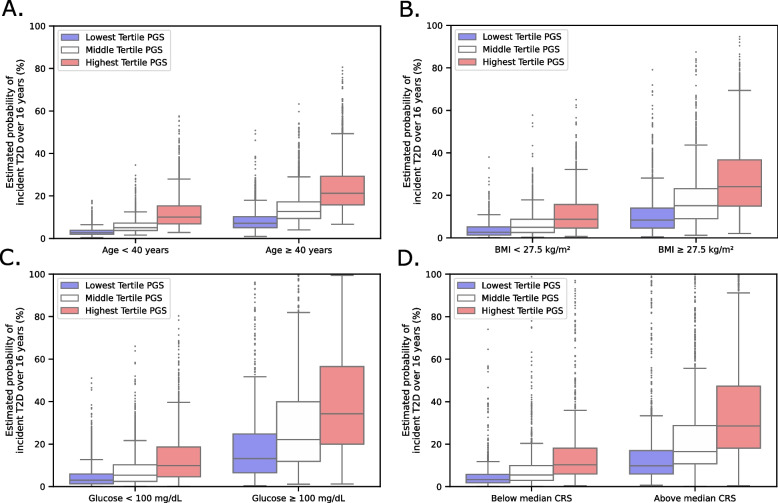
Estimated probability of T2D incidence over 16 years by T2D PGS and clinical risk factors. Patients with low clinical and low genetic risk for T2D have the lowest estimated probability for developing T2D relative to patients with high clinical and high genetic risk. Patients with high clinical risk and low genetic risk also have similar estimated probability for developing T2D compared to patients with low clinical risk and high genetic risk in (**A**) scenario 1, (**B**) scenario 2, (**C**) scenario 3, and (**D**) scenario 4

In addition to hazard ratios, we also calculated each patient’s estimated probability of incident T2D in each scenario. Patients with an age < 40 years in the highest PGS tertile had higher estimated probability (median estimated probability: 10.1% (IQR 6.9%, 15.3%)) compared to patients with an age ≥ 40 years in the lowest PGS tertile (median estimated probability: 7.2% (IQR 5.0%, 10.2%)) (Figs. 2E and 3A). Patients with BMI < 27.5 kg/m^2^ in the highest PGS tertile had similar estimated probability (median estimated probability: 8.7% (IQR 4.6%, 15.7%)) to patients with BMI >27.5 kg/m^2^ in the lowest PGS tertile (median estimated probability: 8.3% (IQR 4.6%, 14.0%)) (Figs. 2F and 3B). Individuals with age ≥ 40 years or BMI ≥ 27.5 kg/m^2^ in the highest PGS tertile had a greater than 25% chance of developing T2D during the follow-up period.

Individuals with glucose < 100 mg/dL had lower estimated probability in all three PGS tertiles (lowest PGS tertile: 3.0% median estimated probability (IQR 1.3%, 5.9%); highest PGS tertile: 9.9% median estimated probability (IQR 4.6%, 18.7%)) compared to those with glucose ≥ 100 mg/dL (lowest PGS tertile: 13.2% median estimated probability (IQR 6.5%, 24.7%); highest PGS tertile: 34.2% median estimated probability (IQR 20.0%, 56.4%)) (Figs. 2G and 3C). Similarly, individuals below the median CRS had similar or lower risk across all three PGS tertiles (lowest tertile: 3.3% median estimated probability (IQR 1.8%, 5.8%); highest tertile: 10.3% median estimated probability (IQR 5.9%, 18.1%)) compared to patients above the median CRS (lowest PGS tertile: 9.8% median estimated probability (IQR 6.0%, 17.0%); highest PGS tertile: 28.6% median estimated probability (IQR 18.1%, 47.3%)) (Figs. 2H and 3D).

For the prediction of incident CAD and CKD, both CAD PGS and CKD PGS modestly improved model performance over their respective CRS in people with and without T2D (Additional file 2: Table S6).

## Discussion

The incorporation of genetic information in the clinician’s toolbox promises to improve disease risk prediction and inform individualized health recommendations, preventive strategies, and medical decisions [33]. Yet, genetic liability, modeled by PGS, has been criticized as having minimal contributions to risk prediction beyond traditional clinical risk factors [12, 34]. We designed four hypothetical scenarios to mimic real-world clinical settings that varied in clinical information availability, and tested whether PGS could add value to longitudinal risk prediction of T2D in an ancestrally and medically diverse patient population with up to 16 years of follow-up time in a PCP network. We showed that PGS significantly improved model performance over available clinical risk factors in all scenarios, with the largest improvements in scenarios with minimal clinical risk factors available. In settings where only age and sex were considered, patients in the top 5% of the T2D PGS had an almost threefold risk of developing T2D compared to those in the IQR and patients in the top 2% had a 3.5-fold risk. In contrast when additionally considering BMI, SBP, family history of T2D, triglycerides, total cholesterol, HDL, and random glucose, those in the 5% had double the risk relative to the IQR of the T2D PGS for developing T2D while those in the top 2% had a 2.5-fold risk for developing T2D. Even when considering individuals of high genetic risk as being in the top 10^th^ percentile, these individuals had a 2.5-fold risk of developing T2D in scenarios adjusting for age and sex and a near twofold risk in scenarios including all available T2D clinical risk factors.

We detected T2D PGS interactions with age, BMI, random glucose, and CRS, suggesting that genetic effects were larger in younger and leaner individuals with normoglycemia that have yet to accumulate T2D-related comorbidities [35]. The probability of developing T2D during follow-up in patients with low baseline clinical risk but high genetic risk was similar to those with high baseline clinical risk. Additionally, patients with a high glucose or CRS and high PGS had a 20% higher chance of developing T2D compared to those with a low PGS. An efficient use of healthcare resources could include targeted screening and preventive interventions in patients with both high clinical and genetic risk. Furthermore, as many clinical risk factors, unlike genetics, are modifiable, patients identified to have high clinical risk despite low genetic risk are probably more likely to benefit from risk factor modifications.

Our work is closely aligned with the mission of the Electronic Medical Records and Genomics (eMERGE) network [36], which aims to improve the combined use of both genetic and EHR data in informing healthcare decisions. We approached the issue of incorporating PGS and EHR data by considering the degree of data sparsity at the time of the initial encounter in the healthcare system, a common scenario in real-world clinical practice yet understudied in previous work. Patients lacking clinical data in the EHR are likely to be younger with few or no established comorbidities, or less engaged in preventive care. As genetic effects were found to be largest when only demographics were considered, PGS could be a powerful tool for encouraging individuals with high genetic risk to undergo a clinical risk assessment.

Our study has limitations. Since the PCP network was not a closed system, incident cases could be missed when patients leave the network. Capture of these cases would still be possible if they had returned to the network during the follow-up time, though the date of capture could be after the actual date of diagnosis, extending the observation time, and biasing our results towards the null. Conversely, we recognize possible contamination by type 1 diabetes being captured by the T2D algorithm, though an overwhelming majority of adult-onset diabetes is type 2. Due to small sample sizes, models generated with the T2D PGS as a categorical variable considering high-risk individuals as being in the top 2% may be underpowered, though we still observe higher hazard ratios of the PGS, and significant clinical risk factor model improvements, compared to high-risk cutoffs of 5% and 10%. Furthermore, we compared these high-risk individuals to the IQR as a measure of the effect of being high-genetic risk for T2D relative to being of average genetic risk for T2D. This choice of reference group could be more interpretable to both patients and clinicians, though further work is required to test the impact of this reference in clinical settings. As waist-to-hip ratio and fasting status for glucose were not captured in EHR, we were unable to include these variables in the T2D CRS. Nevertheless, our study more closely reflects the accuracy and granularity of clinical information captured by EHR. The Framingham T2D CRS was derived from a non-Hispanic White cohort, which may be less accurate in racially/ethnically diverse populations. While we successfully modeled genetic risk in an ancestrally diverse cohort primary care network, most participants were of European ancestry and the PGS performed worse in patients with less than 50% European genetic ancestry. Despite this, we observed the same trend of improved predictive performance when PGS was added to base models composed of only traditional risk factors among patients that were not of European ancestry, indicating that study findings are likely still applicable to more racially and ethnically diverse US-based healthcare systems with longitudinal care. We also acknowledge that patients with existing genetic data in the biobank may not be fully representative of the patients in the network, highlighting the importance of ensuring that access to genetic information is equitably distributed throughout a healthcare system to avoid exacerbating disparities of care of minoritized or marginalized groups that are already disproportionately affected by T2D. We recognize that improvements to the transferability of PGS and replication in other healthcare systems is necessary to properly evaluate the clinical utility of PGS among diverse populations.

## Conclusion

With the growing literature on the predictive accuracy of PGS in diverse populations and the increasing availability of genetic information in healthcare systems, it is becoming crucial to identify when and how PGS can be used in clinical settings. We considered a range of scenarios that varied in clinical information availability to evaluate the role of polygenic risk in T2D prediction in primary care. The utility of PGS for the purpose of identifying high-risk individuals was greatest among those with sparse clinical data and those that were younger, leaner, had few or no established cardiometabolic comorbidities, and may be perceived to have low clinical risk following a clinical evaluation. Considering genetic risk in healthcare systems provides an additional opportunity to engage high-risk patients in preventive strategies and deploy precision medicine approaches in diabetes care.

### Supplementary Information

**Additional file 1:** **Supplementary Methods.** Document containing more in-depth details on the clinical risk scores and Cox models used in the T2D, CAD, and CKD analyses.

**Additional file 2:** **Supplementary Tables.** Excel file containing all six supplemental tables. (1) - Administrative and ICD code based algorithms. (2) - Patient characteristics of the Mass General Hospital PCP network and the Mass General Brigham Biobank. (3) - Association of T2D polygenic scores as a continuous variable with incident T2D. (4) - Association of T2D polygenic scores as a categorical variable (top 10% vs IQR and top 2% vs IQR) with incident T2D. (5) - Association of T2D polygenic scores with incident T2D stratified by clinical risk factors. (6) - Association of polygenic scores with incident coronary artery disease and chronic kidney disease.

**Additional file 3:** **Supplementary Figure S1.** Kaplan-Meier curves of T2D PGS tertiles by genetic similarity to European ancestry.

## Data Availability

Summary statistics used in the calculation of the T2D PGS were obtained from the MVP/DIAMANTE [18] and FINNGEN Biobank r6. The MVP/DIAMANTE summary statistics are available through dbGAP under accession number phs001672.v3.p1. Access to the FINNGEN summary statistics is available through the FINNGEN website (https://www.finngen.fi/en/access_results) [19]. The two summary statistics were then meta-analyzed with inverse-variance weighting together using the publicly available METAL [37] (https://csg.sph.umich.edu/abecasis/Metal/download/). Summary statistics used to calculate the CAD PGS were downloaded from CARDIoGRAM website (http://www.cardiogramplusc4d.org/data-downloads/) [32]. Summary statistics used to calculate the CKD PGS were downloaded from the CKDGen website (http://ckdgen.imbi.uni-freiburg.de/datasets/Wuttke_2019) [31]. PGS were calculated using the publicly available PRScs and 1000G LD panels (https://github.com/getian107/PRScs) [20]. PGS weights are available via the PGS catalog (https://www.pgscatalog.org, publication ID: PGP000619, scores IDs: PGS004887, PGS004888, and PGS004889). The clinical and genetic datasets generated and/or analyzed during the current study are not publicly available due to restrictions that may apply to preserve patient confidentiality but are available from the corresponding author on reasonable request. Summarized data may be shared within 2 weeks from request. Sharing of individual-level data would only be permissible if re-identification of patients is not possible.

## Abbreviations

T2D: Type 2 diabetes
PGS: Polygenic score
BMI: Body mass index
CRS: Clinical risk score
HR: Hazard ratio
SD: Standard deviation
IQR: Interquartile range
PCP: Primary care physician
LRT: Likelihood ratio test
CI: Confidence interval
GWAS: Genome-wide association studies
MGH: Mass General Hospital
MGB: Mass General Brigham
EHR: Electronic health record
HTN: Hypertension
CAD: Coronary artery disease
CKD: Chronic kidney disease
HDL: High-density lipoprotein
MEGA: Illumina Multi-Ethnic Genotyping Array
GSA: Illumina Global Screening Array
PC: Principal Components
SBP: Systolic blood pressure
DBP: Diastolic blood pressure
