# Supplementary material for: Polygenic scores for longitudinal prediction of incident type 2 diabetes in an ancestrally and medically diverse primary care physician network: a patient cohort study
Source: Genome Med. 2024 Apr 26;16:63. doi: 10.1186/s13073-024-01337-0 (PMC11046943; doi:10.1186/s13073-024-01337-0)
Supplement: Supplementary file 1 — Additional file 1: Supplementary Methods. Document containing more in-depth details on the clinical risk scores and Cox models used in the T2D, CAD, and CKD analyses. [file 13073_2024_1337_MOESM1_ESM.docx]

**Supplementary Methods**

**Calculation of T2D, CAD, and CKD CRS**

We implemented the Framingham T2D CRS, comprising age, sex, parental history of T2D, BMI, systolic blood pressure (SBP), high density lipoprotein (HDL), total cholesterol, triglyceride, fasting glucose, and waist circumference. We used family history as a proxy for parental history. As waist circumference and fasting status for glucose were not available, both variables were set as constants in the CRS (100 for waist circumference and 140 for fasting glucose) and random glucose was included as an independent variable. The full CRS implemented was:

$$T2D CRS=age*log (0.99)+{sex}_{female}*log (0.65)+ family history of T2D*log (1.5)+ BMI*log (1.04)+SBP *log (1.01)+HDL *log (0.96)+triglycerides*log (1)+100 *log (1.05)+140 *log (1.15)$$

where *sex_female_* equals 1 if a person reports as being female and 0 if male and *family history of T2D* equals 1 if a person has a family history of T2D. Coefficients were log of the published odds ratios for each variable in the Framingham T2D CRS.

For CAD we used the Framingham CAD CRS, which required age, sex, smoking, total cholesterol, HDL measurements, SBP, and HTN treatment. We used HTN diagnosis as a substitute for HTN treatment. The full CAD CRS used was:

*Men*

$$L_{men}=52.01*log (age)+20.01*log (cholesterol )-0.91*log (HDL)+1.31*\log(SBP)+0.24*HTN+12.10*smoking status+4.61*log (age)*\log(cholesterol)+2.93*log {(age}^{2})-2.84*log {(age}_{70})*smoking status - 172.30$$

$$CAD CRS=1-{0.9402}^{exp(L_{men})}$$

*Women*

$$L_{women}=31.76*log (age)+22.57*log (cholesterol)-1.19*log (HDL)+2.55*log (SBP)+0.42*HTN+13.08*smoking status+5.06*log (age)*\log(cholesterol)-3.00*log {(age}_{70})*smoking status- 146.59$$

$$CAD CRS=1-{0.98767}^{exp(L_{women})}$$

where age_70_ equals age for individuals younger than 70, and equals 70 for individuals older than 70, and *smoking status* equals 1 if a person was a current smoker at baseline.

For CKD we used the SCORED CRS, which required age, sex, and diagnoses of anemia, HTN, diabetes, cardiovascular disease, congestive heart failure, peripheral vascular disease, and proteinuria diagnoses. The full CKD CRS used was:

$$L= -5.4+1.55{*age}_{50-59}+2.31{*age}_{60-69}+3.23*{age}_{70\leq}+0.29{*sex}_{female}+0.93*anemia+0.45*HTN+0.44*diabetes+0.59*CVD+0.45*CHF+ 0.74*PVD+0.83*proteinuria$$

$$CKD CRS=\frac{1}{1+exp(-L)}$$

where *age_50-59_* equals 1 if a person’s age is between 50 and 59, *age_60-69_* equals 1 if a person’s age is between 60 and 69, *age_70≤_* equals 1 if a person’s age is 70 or higher, *sex_female_* equals 1 if a person is a female, *anemia* equals 1 if a person has anemia, *HTN* equals 1 if a person has HTN, *diabetes* equals 1 if a person has diabetes, *CVD* equals 1 if a person has cardiovascular disease, *CHF* equals 1 if a person has congestive heart failure, *PVD* equals 1 if a person has peripheral vascular disease, and *proteinuria* equals 1 if a person has proteinuria. Anemia status was determined based on a hemoglobin count of less than 12 in females and less than 13 in males.

**T2D incidence Cox models**

The T2D Cox model formulas used in each scenario were:

Scenario 1

*PGS only model*

$$Follow up time, T2D status \sim T2D PGS+10 PCs$$

*Clinical Risk Factors only model*

$$Follow up time, T2D status \sim age+{sex}_{female}+ 10 PCs$$

*Combined PGS and Clinical Risk Factor model*

$$Follow up time, T2D status \sim T2D PGS+age+{sex}_{female}+ 10 PCs$$

Scenario 2

*PGS only model*

$$Follow up time, T2D status \sim T2D PGS+10 PCs$$

*Clinical Risk Factors only model*

$$Follow up time, T2D status \sim age+{sex}_{female}+family history of T2D+ BMI+SBP+ 10 PCs$$

*Combined PGS and Clinical Risk Factor model*

$$Follow up time, T2D status \sim T2D PGS+age+{sex}_{female}+ family history of T2D+BMI+SBP+10 PCs$$

Scenario 3

*PGS only model*

$$Follow up time, T2D status \sim T2D PGS+10 PCs$$

*Clinical Risk Factors only model*

$$Follow up time, T2D status \sim age+{sex}_{female}+ family history of T2D+BMI+SBP+glucose+10 PCs$$

*Combined PGS and Clinical Risk Factor model*

$$Follow up time, T2D status \sim T2D PGS+age+{sex}_{female}+ family history of T2D+BMI+SBP+glucose+10 PCs$$

Scenario 4

*PGS only model*

$$Follow up time, T2D status \sim T2D PGS+10 PCs$$

*Clinical Risk Factors only model*

$$Follow up time, T2D status \sim T2D CRS+glucose+ 10 PCs$$

*Combined PGS and Clinical Risk Factor model*

$$Follow up time, T2D status \sim T2D PGS+T2D CRS+glucose+ 10 PCs$$

where sex_female_ equals 1 if a person reports as being female, family history of T2D equals 1 if a person has a family history of T2D, and 10 PCs corresponds to the first 10 PCs.

**CAD incidence Cox models**

The CAD Cox model formulas used in each scenario were:

Clinical Visit Scenario

*PGS only model*

$$Follow up time, CAD status \sim CAD PGS+10 PCs$$

*Clinical Risk Factors only model*

$$Follow up time, CAD status \sim age+{sex}_{female}+smoking status+SBP+ 10 PCs$$

*Combined PGS and Clinical Risk Factor model*

$$Follow up time, CAD status \sim CAD PGS+age+{sex}_{female}+smoking status+SBP+ 10 PCs$$

Clinical Visit with Labs Scenario

*PGS only model*

$$Follow up time, CAD status \sim CAD PGS+10 PCs$$

*Clinical Risk Factors only model*

$$Follow up time, CAD status \sim CAD CRS+ 10 PCs$$

*Combined PGS and Clinical Risk Factor model*

$$Follow up time, CAD status \sim CAD PGS+CAD CRS+ 10 PCs$$

**CKD incidence Cox models**

The CKD Cox model formulas used in each scenario were:

Clinical Visit Scenario

*PGS only model*

$$Follow up time, CKD status \sim CKD PGS+10 PCs$$

*Clinical Risk Factors only model*

$$Follow up time, CKD status \sim age+{sex}_{female}+SBP+ DBP+weight+HTN+proteinuria+ CVD+CHF+PVD+10 PCs$$

*Combined PGS and Clinical Risk Factor model*

$$Follow up time, CKD status \sim CKD PGS+age+{sex}_{female}+SBP+ DBP+weight+HTN+proteinuria+ CVD+CHF+PVD+ 10 PCs$$

Clinical Visit with Labs Scenario

*PGS only model*

$$Follow up time, CKD status \sim CKD PGS+10 PCs$$

*Clinical Risk Factors only model*

$$Follow up time, CKD status \sim CKD CRS+ 10 PCs$$

*Combined PGS and Clinical Risk Factor model*

$$Follow up time, CKD status \sim CKD PGS+CKD CRS+ 10 PCs$$
