# Supplementary material for: Polygenic scores for longitudinal prediction of incident type 2 diabetes in an ancestrally and medically diverse primary care physician network: a patient cohort study
Source: Genome Med. 2024 Apr 26;16:63. doi: 10.1186/s13073-024-01337-0 (PMC11046943; doi:10.1186/s13073-024-01337-0)
Supplement: Supplementary file 3 — Additional file 3: Supplementary Figure S1. Kaplan-Meier curves of T2D PGS tertiles by genetic similarity to European ancestry. [file 13073_2024_1337_MOESM3_ESM.docx]

**Fig. S1:** **Kaplan-meier curves of T2D PGS tertiles by genetic similarity to European ancestry**.


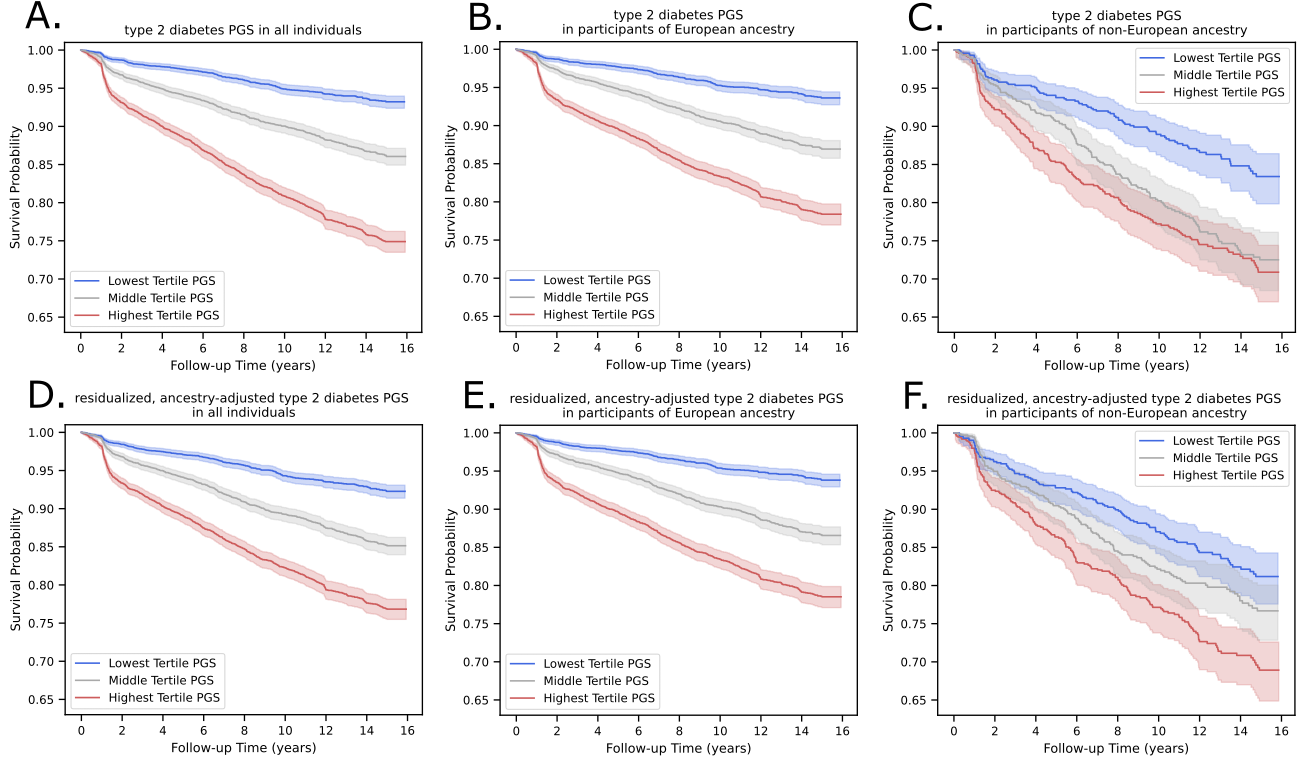


Within (A) the total population and (B) a subset of individuals of only EUR ancestry, T2D PGS tertiles separate risk of diabetes onset. However in (C) individuals of non-EUR ancestry, the highest and middle T2D PGS tertiles overlap. Residualizing ancestry bias from the T2D PGS maintains similar separation within (D) the total population and (E) participants of EUR ancestry. (F) Residualization further improves T2D PGS tertile separation in non-EUR participants. Multivariate log-rank test *P* in all analyses was <0.0001.
